# Δ40p53α suppresses tumor cell proliferation and induces cellular senescence in hepatocellular carcinoma cells

**DOI:** 10.1242/jcs.190736

**Published:** 2017-02-01

**Authors:** Akinobu Ota, Haruhisa Nakao, Yumi Sawada, Sivasundaram Karnan, Md Wahiduzzaman, Tadahisa Inoue, Yuji Kobayashi, Takaya Yamamoto, Norimitsu Ishii, Tomohiko Ohashi, Yukiomi Nakade, Ken Sato, Kiyoaki Itoh, Hiroyuki Konishi, Yoshitaka Hosokawa, Masashi Yoneda

**Affiliations:** 1 Department of Biochemistry, Aichi Medical University School of Medicine, Nagakute, Aichi, Japan; 2 Division of Hepatology and Pancreatology, Department of Internal Medicine, Aichi Medical University School of Medicine, Nagakute, Aichi, Japan

**Keywords:** Δ40p53, Gene targeting, Liver cancer, Tumorigenesis, Gene expression

## Abstract

Splice variants of certain genes impact on genetic biodiversity in mammals. The tumor suppressor *TP53* gene (encoding p53) plays an important role in the regulation of tumorigenesis in hepatocellular carcinoma (HCC). Δ40p53α is a naturally occurring p53 isoform that lacks the N-terminal transactivation domain, yet little is known about the role of Δ40p53α in the development of HCC. Here, we first report on the role of Δ40p53α in HCC cell lines. In the *TP53^+/Δ40^* cell clones, clonogenic activity and cell survival dramatically decreased, whereas the percentage of senescence-associated β-galactosidase (SA-β-gal)-positive cells and p21 (also known as WAF1, CIP1 and CDKN1A) expression significantly increased. These observations were clearly attenuated in the *TP53^+/Δ40^* cell clones after Δ40p53α knockdown. In addition, exogenous Δ40p53 expression significantly suppressed cell growth in HCC cells with wild-type *TP53*, and in those that were mutant or null for *TP53*. Notably, Δ40p53α-induced tumor suppressor activity was markedly attenuated in cells expressing the hot-spot mutant Δ40p53α-R175H, which lacks the transcription factor activity of p53. Moreover, Δ40p53α expression was associated with increased full-length p53 protein expression. These findings enhance the understanding of the molecular pathogenesis of HCC and show that Δ40p53α acts as an important tumor suppressor in HCC cells.

## Abstract (precis)

**Summary:** Δ40p53 exerts tumor suppressor activity that is associated with upregulation of p53-target gene expression and induces senescence in hepatocellular carcinoma cell lines.

## INTRODUCTION

Hepatocellular carcinoma (HCC), one of the most frequent malignancies worldwide (Lozano et al., 2012), commonly develops in response to continuous microenvironmental stresses, including chemical exposure, chronic inflammation from hepatitis viral infection, tissue remodeling in the liver, and a high-fat diet (Liu et al., 2014; Nishida and Goel, 2011). As an initiating oncogenic event in HCC, the disruption of the *TP53* tumor suppressor gene (encoding p53) has been shown to be closely associated with hepatocarcinogenesis. Deletion of the equivalent gene in mice, *Trp53*, results in the development of liver tumors in a significant number of mice (Katz et al., 2012; Morris et al., 2012), while restoring p53 in a murine liver carcinoma model limits tumor cell growth by mediating cellular senescence (Xue et al., 2007). Thus, accumulating evidence implicates *TP53* gene dysfunction in the development of HCC.

In general, splice variants of certain genes play an important role in biodiversity. It is known that the *TP53* gene potentially encodes at least 12 p53 isoforms, in which four different N-terminal p53 forms (full-length, Δ40, Δ133 and Δ160) are combined with three different C-terminal domains (α, β and γ) (Marcel et al., 2011). Full-length (FL)-p53 protein (also called TAp53α) is the canonical p53 protein, while Δ40p53α (also known as p53 or p47), a p53 isoform that lacks the 39 N-terminal amino acids corresponding to the first transactivation domain (TAD-I) of FL-p53, is translated from an in-frame second AUG at nucleotides 252–254 of *TP53* mRNA through a second internal ribosome entry site (Olivares-Illana and Fåhraeus et al., 2010; Wei et al., 2012). Recent studies demonstrated the biological effects of Δ40p53α in both humans and mice. Transgenic mice overexpressing p44, the mouse homolog of Δ40p53α, showed obvious signs of aging and a shorter lifespan (Maier et al., 2004; Qian and Chen, 2013). It has been reported that Δ40p53α exerts anti-cancer properties in human lung cancer and melanoma cells (Yin et al., 2002; Candeias et al., 2006; Takahashi et al., 2014). In contrast, Courtois et al. reported that Δ40p53α counteracts growth suppression via FL-p53 in mouse fibroblasts (Courtois et al., 2002). Thus, the biological function of Δ40p53α potentially varies according to cell type. Although accumulating evidence has implicated Δ40p53α in aging and/or tumor suppression, little is known about the involvement of Δ40p53α in the development of HCC.

In the present study, we are the first to report the tumor suppressor role of Δ40p53α (hereafter called Δ40p53) in the development of HCC. We also discuss a possible molecular mechanism underlying Δ40p53-induced tumor suppression and senescence.

## RESULTS

### Establishment of *TP53^+/Δ40^* HepG2 cell clones expressing Δ40p53

**Fig. 1.**
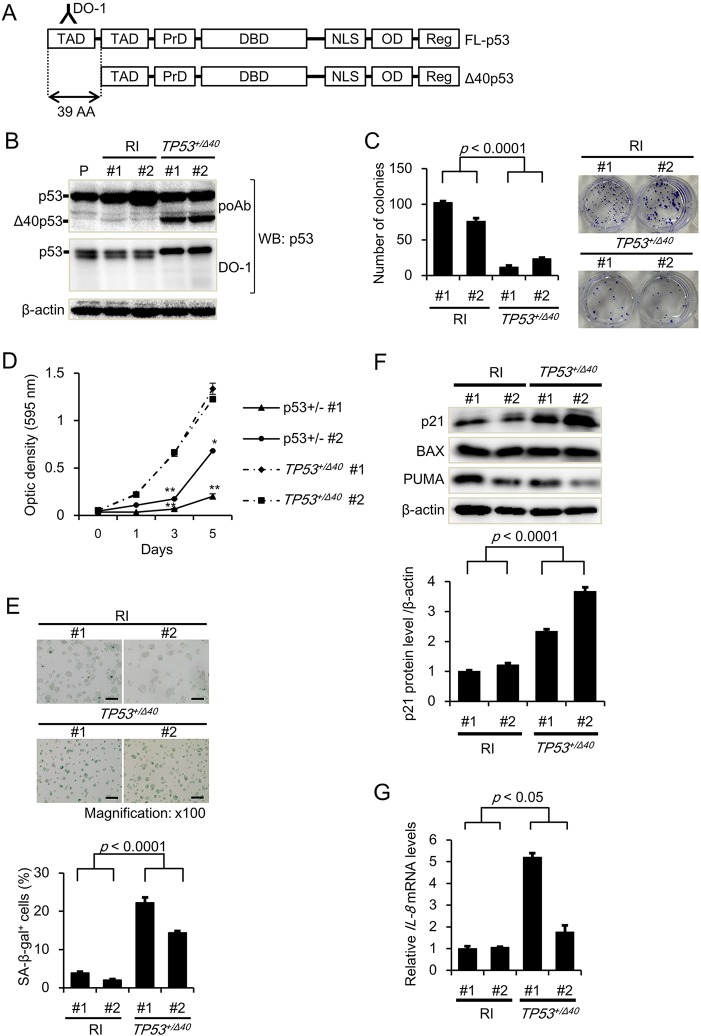
**The cellular phenotype of *TP53^+/Δ40^* HepG2 cells.** (A) A schematic of the domain structures of the human p53 protein and the Δ40p53 isoform. A monoclonal antibody, DO-1, recognizes the first TAD domain in p53 that is not present in Δ40p53. TAD, transactivation domain; PrD, proline-rich domain; DBD, DNA-binding domain; NLS, nuclear localization signal; OD, oligomerization domain; Reg, regulatory domain. (B) p53 protein levels were examined by western blot (WB) analysis. We analyzed 5 μg of cell lysate by western blotting to detect p53 isoforms with an anti-p53 polyclonal antibody (poAb) or an anti-p53 monoclonal antibody DO-1. The HepG2 parental cells (P), RI clones (#1 and #2), and *TP53^+/Δ40^* clones (#1 and #2) are shown. β-actin was used as an internal control. (C) A representative colony formation assay with HepG2 RI and *TP53^+/Δ40^* clones; 200 were seeded in a 6-well plate. After 14 days, the cells were stained with Crystal Violet and imaged. Bar graphs represent the number of stained colonies. Data are presented as the mean±s.e.m. (*n*=6). (D) MTT analysis of the growth rate of HepG2 RI and *TP53^+/Δ40^* clones. The optical density (595 nm) at each time point (day 0, 1, 3 and 5) is presented as the mean±s.e.m. (*n*=4). *P<0.05; **P<0.005 compared to RI clone #1. (E) Analysis of cellular senescence using the SA-β-gal assay; 20,000 cells were seeded in 12-well plates, and then incubated for 48 h. After incubation, the cells were stained for SA-β-gal to determine the β-gal activity. Representative result of SA-β-gal staining (taken at a magnification of ×100) is shown above. Bar graphs represent the percentage of SA-β-gal-positive cells. Data are presented as the mean±s.e.m. (*n*=3). Scale bar: 200 µm. (F) Protein expression of p21, BAX, and PUMA. We analyzed 5 μg of the protein by western blotting with an antibody specific to p21, BAX, and PUMA. β-actin was used as an internal control. After normalization to β-actin protein levels, the protein levels of p21 are expressed relative to the protein expression in the HepG2/RI #1 cells, which was arbitrarily defined as 1. The data are represented as the mean±s.e.m. of three separate experiments. (G) qRT-PCR analysis of the gene expression levels of *IL-8* in RI clone and HepG2/*TP53^+/Δ40^* clones. The primers used for qRT-PCR are shown in Table S3. Relative gene expression levels are shown after normalization to *GAPDH* mRNA expression. The data are expressed relative to the mRNA levels found in the corresponding sample of HepG2/RI clone #1 cells, which was arbitrarily defined as 1. The values shown represent the mean±s.e.m. (*n*=3).

We first performed gene targeting of wild-type (WT) *TP53* in the human HepG2 hepatoma cell line and generated isogenic cell clones harboring exon 2 deletions of *TP53* to induce endogenous Δ40p53 expression using adeno-associated virus (AAV)-based methodology (Fig. S1A), as previously observed in colon cancer HCT116 *TP53*^−/−^ cell clones previously (Thomas et al., 2013). We established two independent heterozygous exon 2-deleted *TP53* HepG2 cell clones (denoted *TP53^+/Δ40^* #1 and #2). Gene targeting was successfully confirmed by PCR amplification of the targeted genomic locus (Fig. S1B). In addition, we isolated cell clones that underwent random integration (RI) of the targeting vectors within their genomes (RI #1 and #2); these clones were used as controls for the *TP53^+/Δ40^* clones. Fig. 1A shows a schematic of the FL-p53 and Δ40p53 protein domains, illustrating the lack of an N-terminal TAD-I domain (corresponding to FL-p53 residues 1–39) in Δ40p53. We next examined the protein expression of the p53 isoforms by western blot analysis and determined that an anti-p53 polyclonal antibody (pAb) that recognizes both isoforms clearly detected Δ40p53 protein in the *TP53^+/Δ40^* clones but not in the RI clones, whereas an anti-p53 monoclonal antibody (mAb; DO-1) that recognizes residues 11–25, which are present only in FL-p53, did not detect Δ40p53 protein in the *TP53^+/Δ40^* clones (Fig. 1B). We confirmed that the molecular mass of Δ40p53 in the *TP53^+/Δ40^* clones was almost identical to that of Δ40p53 exogenously expressed via retrovirus in HepG2 cells (Fig. S1C). These results indicate that the shorter p53 isoform in the *TP53^+/Δ40^* clones is most likely the Δ40p53 protein. We next attempted to create *p53^Δ40/Δ40^* cell clones by targeting the remaining WT allele in *TP53^+/Δ40^* clones. However, despite several attempts, we failed to obtain *TP53^Δ40/Δ40^* cells after gene targeting in *TP53^+/Δ40^* cells; all the candidate clones were genotyped as *TP53^+/Δ40^* by genomic PCR amplification (Table S3). Because the lack of the TAD-I domain enables Δ40p53 to avoid MDM2-induced protein degradation (Hafsi et al., 2013), it is a reasonable that an increase in Δ40p53 isoform expression potentially robustly induces cell death and/or growth arrest in null clones.

### Impact of Δ40p53 on tumor cell growth and senescence

To address the biological function of Δ40p53 in the HepG2 cell clones, we performed clonogenicity and MTT assays. As shown in Fig. 1C,D, both colony formation and cell survival were significantly reduced in the *TP53^+/Δ40^* clones compared to the RI clones, suggesting that tumor cell growth was suppressed in the *TP53^+/Δ40^* clones. Transgenic mice overexpressing Δ40p53 were reported to exhibit a striking growth defect with a reduced lifespan and accelerated aging (Maier et al., 2004; Qian and Chen, 2013). These reports prompted us to utilize a β-galactosidase assay to investigate whether cellular senescence is accelerated in *TP53^+/Δ40^* clones. As shown in Fig. 1E, the percentage of senescence-associated β-galactosidase (SA-β-gal)-positive cells was significantly higher in the *TP53^+/Δ40^* clones compared to the RI clones. The increase in SA-β-gal-positive cells was continuously observed in the *TP53^+/Δ40^* clones. In addition, western blot analysis revealed that protein expression of p21 (also known as WAF1, CIP1 and CDKN1A), but not of BAX and PUMA (also known as BBC3), significantly increased in *TP53^+/Δ40^* clones (Fig. 1F). We also found that caspase-3 and -7 activity and the percentage of apoptotic cells did not significantly increase in the *TP53^+/Δ40^* clones compared to the RI clones (Fig. S2A,B), suggesting that the growth suppression was not a result of increased apoptosis. Thus, we next examined that mRNA expression of *IL-8*, one of the prominent components of the senescence-associated secretory phenotype (SASP), using quantitative real-time PCR (qRT-PCR) analysis. As shown in Fig. 1G, mRNA expression of *IL-8* significantly increased in *TP53^+/Δ40^* clones. Furthermore, the mRNA levels of p53-induced genes, including *p21*, *MDM2* and *FAS* were significantly increased in *TP53^+/Δ40^* clones compared to the RI clones (Fig. S2C,D). These results indicate that cellular senescence is accelerated in the *TP53^+/Δ40^* clones.

### Promising tumor suppressor activity of Δ40p53 in *TP53*-knockdown conditions

**Fig. 2.**
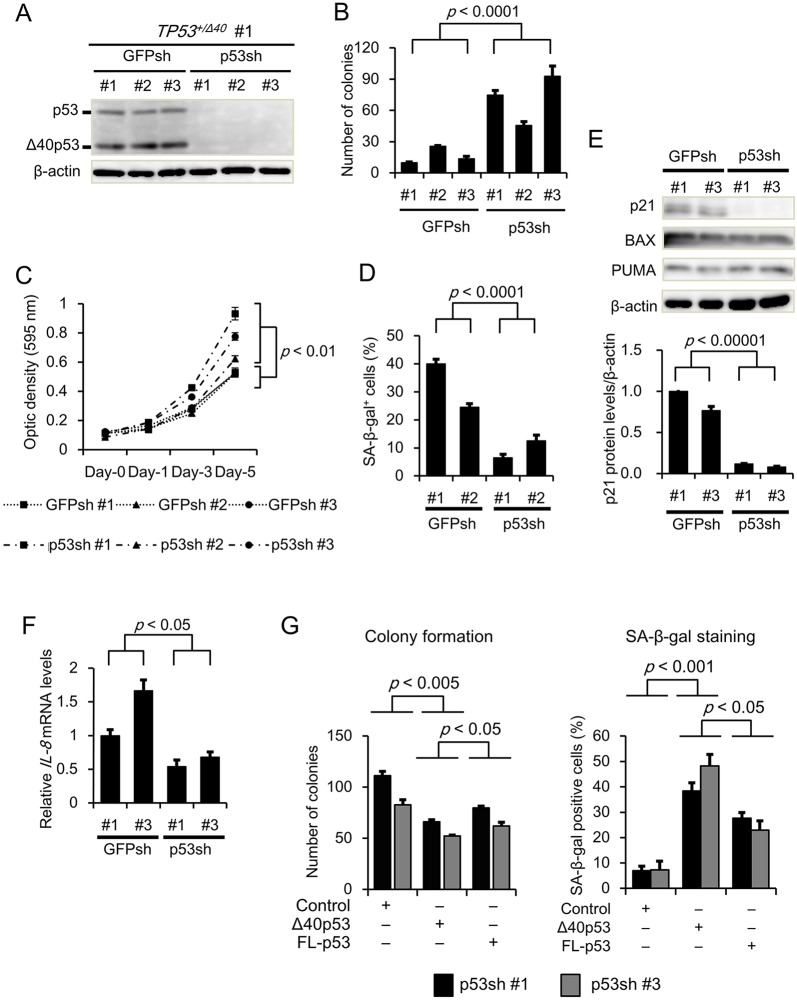
**Effect of *TP53* gene knockdown on cell growth and senescence in HepG2/*TP53^+/Δ40^* cells.** (A) Confirmation of *TP53* gene knockdown in HepG2/*TP53^+/Δ40^* cells. Protein levels of p53 isoforms were examined by western blotting with an anti-p53 polyclonal antibody. The HepG2/*TP53^+/Δ40^*/GFPsh clones (#1 to #3) and HepG2/*TP53^+/Δ40^*/p53sh clones (#1 to #3) are shown. β-actin was used as an internal control. (B) Effect of *TP53* gene knockdown on clonogenicity. Colony formation assays were performed as described in the legend of Fig. 1C. Bar graphs represent the number of stained colonies (*n*=6). (C) Effect of *TP53* gene knockdown on cell survival. An MTT assay was performed as described in the legend of Fig. 1D. The optical density (595 nm) at each time point (day 0, 1, 3, and 5) is expressed as the mean±s.e.m. (*n*=4). (D) Effect of *TP53* gene knockdown on cellular senescence. A SA-β-gal assay was performed as described in the legend of Fig. 1E. Bar graphs represent the percentage of SA-β-gal-positive cells (*n*=3). (E) Protein expression of p21, BAX and PUMA were examined by western blotting as described in the legend of Fig. 1F. After normalization to β-actin protein levels, the data are expressed relative to the protein expression in the HepG2/*TP53^+/Δ40^*/GFPsh #1 cells, which was arbitrarily defined as 1. (F) qRT-PCR analysis for the gene expression levels of *IL-8* in HepG2/GFPsh and HepG2/p53sh clones. Relative gene expression levels are shown after normalization to *GAPDH* mRNA expression. The data are expressed relative to the mRNA level found in the corresponding sample of HepG2/GFPsh clone #1, which was arbitrarily defined as 1. (G) Effect of Δ40p53 on clonogenicity and senescence in the HepG2/*TP53^+/Δ40^*/p53sh clones. pLXSN, Δ40p53/PLXSN and FL-p53/PLXSN retroviruses were generated using 293T cells. After the viral supernatants were prepared, the HepG2/*TP53^+/Δ40^*/p53sh clones (#1 and #3) were infected at the same MOI with pLXSN, Δ40p53/PLXSN, and FL-p53. After infection for 48 h, the cells were treated with neomycin (800 μg/ml) for 14 days, followed by staining with either Crystal Violet or for SA-β-gal. The upper and lower bar graphs represent the number of colonies and the percentage of SA-β-gal-positive cells, respectively (mean±s.e.m.; *n*=3).

To further investigate the tumor suppressor activity of Δ40p53 in HepG2 clones, we examined clonogenic activity, cellular growth and SA-β-gal activity in *TP53^+/Δ40^* cells after knocking down the *TP53* gene using retrovirus-mediated RNA interference (RNAi). We established three *TP53*-knockdown clones (p53sh #1, #2, and #3) and control (*GFP*) clones (GFPsh #1, #2, and #3) from the parental *TP53^+/Δ40^* #1 cell clone. We confirmed that the expression of p53 isoforms at both the mRNA and protein levels was markedly reduced in all the p53sh clones compared to the GFPsh clones (Fig. S3A; Fig. 2A). Next, we investigated the effect of p53 knockdown on clonogenic activity using a colony formation assay. Colony formation was significantly increased in the p53sh clones compared to the GFPsh clones (Fig. 2B). Similarly, cell survival was significantly increased in the p53sh clones compared to GFPsh clones (Fig. 2C). In addition, p53 knockdown significantly decreased the percentage of SA-β-gal-positive cells (Fig. 2D) and p21 mRNA and protein expression, but not BAX and PUMA protein expression (Fig. S3B; Fig. 2E). qRT-PCR analysis revealed that the mRNA levels of *MDM2*, *GADD45A* and *FAS*, but not of *BAX*, *PUMA* and *CCNB1*, were significantly decreased in the p53sh clones compared with the GFPsh clones (Fig. S3C). Based on these results and data demonstrating that the induction of *p21* gene expression results in G1-phase arrest, we examined the cell cycle distribution in p53sh clones using propidium iodide (PI) staining-based cell cycle analysis (Ozturk et al., 2009; Larsson, 2011). The population of cells in G1 decreased in response to p53 knockdown (Fig. S3D). Furthermore, we rescued Δ40p53 expression in the p53sh #1 and #3 clones using a retroviral expression vector. Rescuing Δ40p53 expression reversed the increase in colony formation and the decrease in the percentage of SA-β-gal-positive cells (Fig. 2G). We observed that rescuing Δ40p53 expression did not affect the result of colony formation in the GFPsh clones (data not shown). These results strongly suggest that Δ40p53 suppresses tumor cell growth and promotes cellular senescence.

### Exogenous expression of Δ40p53 suppresses cell growth and enhances senescence and G_1_ arrest

**Fig. 3.**
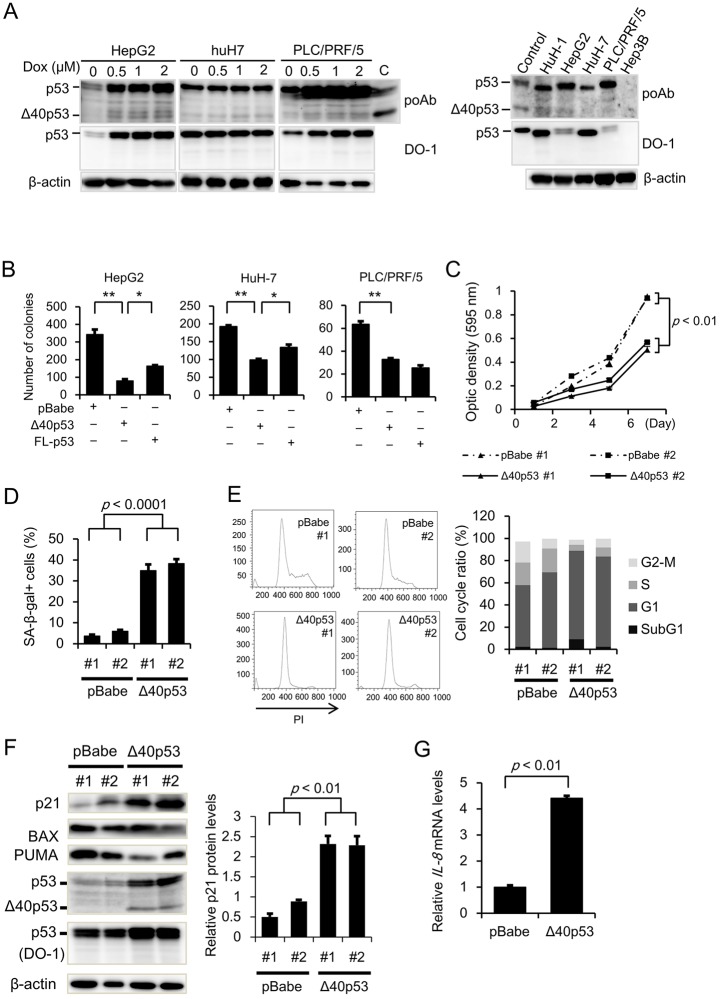
**Effect of exogenous Δ40p53 expression on cell growth and senescence.** (A) Protein expression levels of Δ40p53 in HCC cells. Left panel, inducible protein expression of Δ40p53. HepG2, PLC/PRF/5, and HuH-7 cells were incubated in medium containing the indicated concentration (0, 0.5, 1, 2 μM) of doxorubicin (Dox) for 12 h; right panel, endogenous protein expression of Δ40p53 in HCC cell lines including HuH-1 (*TP53*^WT^), HepG2 (*TP53*^WT^), PLC/PRF/5 (*TP53*^R249S^), HuH-7 (*TP53*^Y220C^), and Hep3B (*TP53^−/−^*) cells. The cells were lysed with lysis buffer; 5 or 1 μg of protein lysate was then subjected to western blot analysis to detect p53 or β-actin protein, respectively. An anti-p53 polyclonal antibody (poAb) and an anti-p53 monoclonal antibody (DO-1) were used to detect both FL-p53 and Δ40p53. C, control (HepG2 *TP53^+/Δ40^* cells). (B) Effect of Δ40p53 on clonogenicity. pBabe, Δ40p53/pBabe, and FL-p53/pBabe retroviruses were generated using 293T cells. After the viral supernatants were prepared, HepG2 cells, PLC/PRF/5, and HuH-7 cells were infected at the same MOI with pBabe, Δ40p53/pBabe, or FL-p53/pBabe. After infection for 48 h, the cells were treated with puromycin (2 μg/ml) for 14 days followed by staining with Crystal Violet and imaging. Bar graphs represent the number of stained colonies (*n*=6). *P<0.05; **P<0.005. (C) The growth rate of the pBabe and Δ40p53/pBabe HepG2 cell clones were determined by using an MTT assay. Each single clone was picked from a dish after 14 days of puromycin treatment, expanded, and then utilized for the following assay. The optical density (595 nm) at each time point (day 0, 2, 4, and 6) is presented as the mean±s.e.m. (*n*=4). (D) The cellular senescence of the pBabe and Δ40p53/pBabe clones was examined as described in the legend of Fig. 1E. Bar graphs represent the percentage of SA-β-gal-positive cells (mean±s.e.m.; *n*=3). (E) Cell cycle analysis of pBabe and Δ40p53/pBabe clones. Each cell clone (10^5^ cells/well) was incubated in serum-free medium for 48 h to synchronize the cell cycle. After 48 h of serum starvation, the cells were further cultured in medium containing 10% FBS for 24 h. The cells were detached, fixed and stained with propidium iodide (PI, 100 μg/ml) after RNase (1 mg/ml) treatment. The cell cycle populations were measured by FACS. A representative FACS histogram and bar graphs of the cell cycle ratios are shown. (F) Protein levels of p21, BAX, PUMA, Δ40p53 and FL-p53 in the pBabe and Δ40p53/pBabe clones were determined by western blot analysis as described in the legend of Fig. 1F. (G) qRT-PCR analysis of relative *IL-8* gene expression level in pBabe and Δ40p53 clones. The data are expressed relative to the mRNA levels found in the corresponding sample of pBabe clones, which was arbitrarily defined as 1.

Given our experimental results in *TP53^+/Δ40^* cells, we next examined Δ40p53 protein expression in HCC cells by western blot analysis. We found that an anti-p53 pAb, but not a DO-1 mAb, detected Δ40p53 protein expression in HepG2, HuH-7, and PLC/PRF/5 cells; these protein levels were further upregulated after treatment with doxorubicin (Fig. 3A, left panel). Therefore, we examined the endogenous Δ40p53 protein expression in HCC cell lines by using western blot analysis. We found that Δ40p53 protein expression was detected in all the HCC cell lines except for the *TP53*-deleted cell line Hep3B (Fig. 3A, right panel). Thus, we examined whether the exogenous expression of Δ40p53 affects tumor cell growth and/or cellular senescence. In a colony formation assay, HepG2 cells expressing Δ40p53 formed fewer colonies than those expressing the control vector (pBabe) or FL-p53 (Fig. 3B). Similarly, clonogenicity was significantly suppressed in both PLC/PRF/5 and HuH-7 cells (Fig. 3B). We established two independent cell clones that exogenously expressed Δ40p53 protein (Δ40p53 #1 and #2) as well as vector control clones (pBabe #1 and #2) from parental HepG2 cells. An MTT assay revealed that cell survival was significantly suppressed in the Δ40p53 clones compared to the pBabe clones (Fig. 3C). In addition, the percentage of SA-β-gal-positive cells was significantly higher for the Δ40p53 clones compared to the pBabe clones (Fig. 3D). These results provide further experimental evidence that Δ40p53 plays a pivotal role in tumor cell growth and senescence. We next examined the effect of Δ40p53 on cell cycle progression. Cell cycle analyses revealed that the population of cells in G1 was larger for the Δ40p53 clones (Fig. 3E), and these clones were desensitized to serum-induced cell cycle progression (data not shown). These results suggest that Δ40p53 may block cell cycle progression at G1. We then examined the effect of Δ40p53 on p53 transcription factor activity (as monitored by a Luciferase-based system) and TP53-induced gene expression, and found that the mRNA and protein expression of p21 as well as the p53 transcription factor activity was significantly increased in the Δ40p53 cell clones (Fig. 3F; Fig. S4A,B). Similarly, mRNA expression level of *IL-8* significantly increased in Δ40p53 cell clone (Fig. 3G). Unexpectedly, FL-p53 protein expression was found to be higher in the Δ40p53 clones compared to the pBabe clones (Fig. 3F, see below). qRT-PCR analysis showed that the mRNA expression of *MDM2* and *FAS*, but not of *BAX*, *PUMA*, *GADD45A* and *CCNB1*, was significantly increased in the Δ40p53 clones compared to the pBabe clones (Fig. S4C), indicating that Δ40p53 exerts tumor suppressor activity by promoting *TP53*-induced gene expression.

### Tumor suppressor activity of Δ40p53 and its mutants

**Fig. 4.**
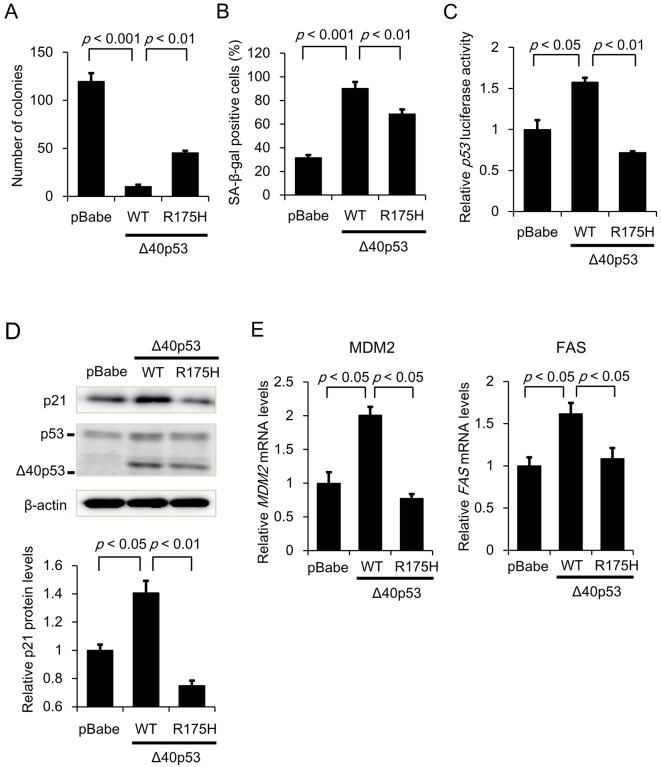
**Involvement of transcriptional activity in the tumor suppressor activity of Δ40p53.** (A) Colony formation assay. pBabe, Δ40p53 (WT)/pBabe, and Δ40p53 (R175H)/pBabe retroviruses were generated using 293T cells. After the viral supernatants were prepared, HepG2 cells were infected as described in the legend of Fig. 3B. Bar graphs represent the number of stained colonies (mean±s.e.m.; *n*=6). (B) SA-β-gal assay. pBabe/HepG2, Δ40p53 (WT)/HepG2, and Δ40p53 (R175H)/HepG2 cells (2×10^4^ cells/well) were seeded in 12-well plates. The cells were incubated for 48 h and then stained. Bar graphs represent the percentage of SA-β-gal-positive cells (mean±s.e.m.; *n*=3). (C) p53-dependent transactivation was examined using a p53 luciferase reporter assay. Each clone was co-transfected with 0.15 μg of the TG13-Luc vector (containing the WT p53 DNA-binding site; *p53* firefly activity) and 0.03 μg of the phRL-TK vector (internal control; *Renilla* luciferase activity). Luciferase activity was measured 48 h after the transfection. After normalization to *Renilla* luciferase activity, the data are expressed relative to the p53-dependent luciferase activity in the HepG2/RI #1 cells, which was arbitrarily defined as 1 (mean±s.e.m.; *n*=4). (D) Protein levels of p21 and p53 isoforms were examined by western blot analysis as described in Fig. 1F. β-actin was used as an internal control. (E) qRT-PCR analysis of *MDM2* and *FAS* gene expression. The relative gene expression levels are shown after normalization to *GAPDH* mRNA expression. Data are presented relative to the mRNA expression in pBabe/HepG2 cells, which was arbitrarily defined as 1 (mean±s.e.m.; *n*=3).

It has been reported that *TP53* gene mutations impair p53-mediated apoptosis and/or p53 transcription factor activity (Serrano et al., 1997; Cui et al., 2002). Thus, we decided to ascertain the effect of Δ40p53 harboring a *TP53* hot-spot missense mutation, R175H, on clonogenic activity and senescence in HepG2 cells. The clonogenicity assay revealed that cells expressing WT Δ40p53 formed fewer colonies; this reduction was significantly attenuated in cells expressing Δ40p53-R175H (Fig. 4A). In addition, the percentage of SA-β-gal-positive cells was significantly higher among those expressing WT Δ40p53; this increase was attenuated in cells expressing Δ40p53-R175H (Fig. 4B). We confirmed that both p53 transcription factor activity and p21 protein expression were markedly increased in cells expressing WT-Δ40p53 compared to control cells, but was not increased in those expressing Δ40p53-R175H (Fig. 4C,D). Furthermore, qRT-PCR analysis demonstrated that Δ40p53-R175H failed to increase the mRNA levels of *MDM2* and *FAS* (Fig. 4E). These results suggest that Δ40p53-induced transcription is closely associated with the roles of Δ40p53 in tumor suppression and/or cellular senescence.

### Δ40p53 itself exerts tumor suppressor activity in *TP53*-deleted cells

**Fig. 5.**
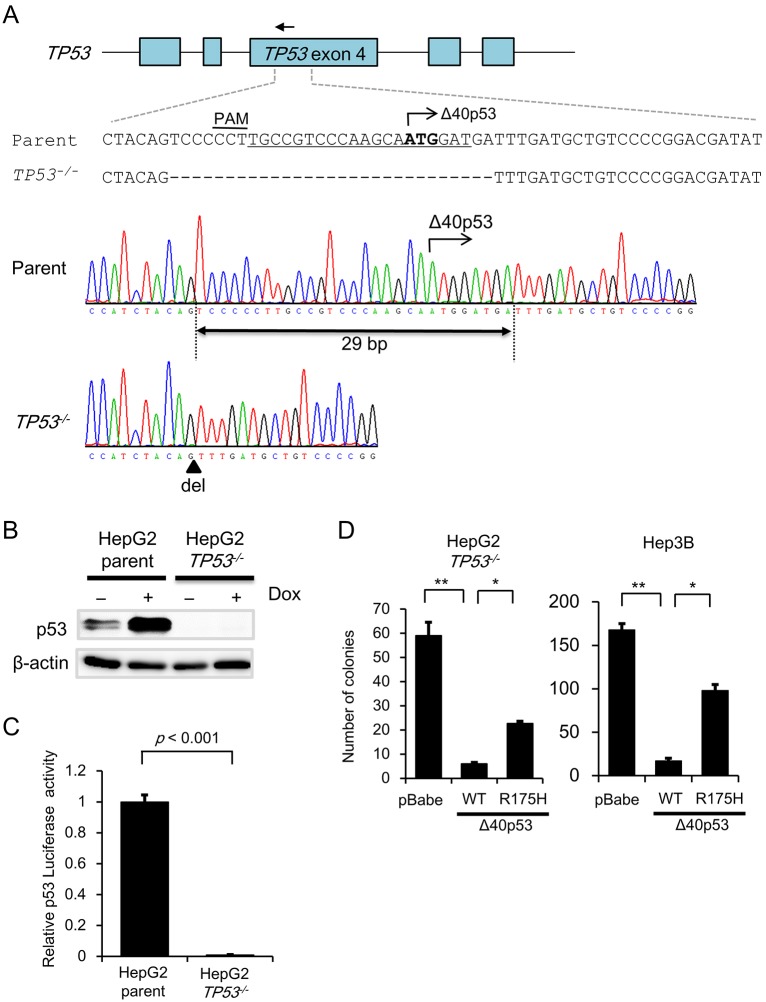
**Effect of Δ40p53 on *TP53*^−/−^ cell growth.** (A) An sgRNA (arrow) sequence was designed against the *TP53* loci to excise an in-frame second ATG (bold) that initiates the transcription of Δ40p53. The sgRNA sequence and the PAM sequence are indicated by underlining and an overbar, respectively. The sequences of the parental and *TP53*^−/−^ (null) cell clones (bi-allelic modification) were analyzed, and the results are shown below. A junction in the *TP53^−/−^* cell clone is indicated by the arrowhead. (B) p53 protein expression was determined by western blot analysis. Parental HepG2 and HepG2/*TP53^−/−^* cells were incubated for 24 h in the presence or absence of doxorubicin (500 nM) and then lysed with lysis buffer; 5 μg of lysate was subjected to western blot analysis to detect p53 protein. β-actin was used as an internal control. (C) p53-dependent transactivation was examined using a p53 luciferase reporter assay as described in Fig. 4C. Luciferase activity was measured 48 h after the transfection. After normalization to *Renilla* luciferase activity, the data are expressed relative to the p53-dependent luciferase activity in the parental HepG2 cells, which was arbitrarily defined as 1 (mean±s.e.m.; *n*=6). (D) Colony formation assay. pBabe, Δ40p53 (WT)/pBabe and Δ40p53 (R175H)/pBabe retroviruses were prepared using 293 T cells. After the viral supernatants were prepared, HepG2/*TP53^−/−^* cells and Hep3B (p53 null) cells were infected as described in the legend of Fig. 4B. Bar graphs represent the number of stained colonies (mean±s.e.m.; *n*=3).

**Fig. 6.**
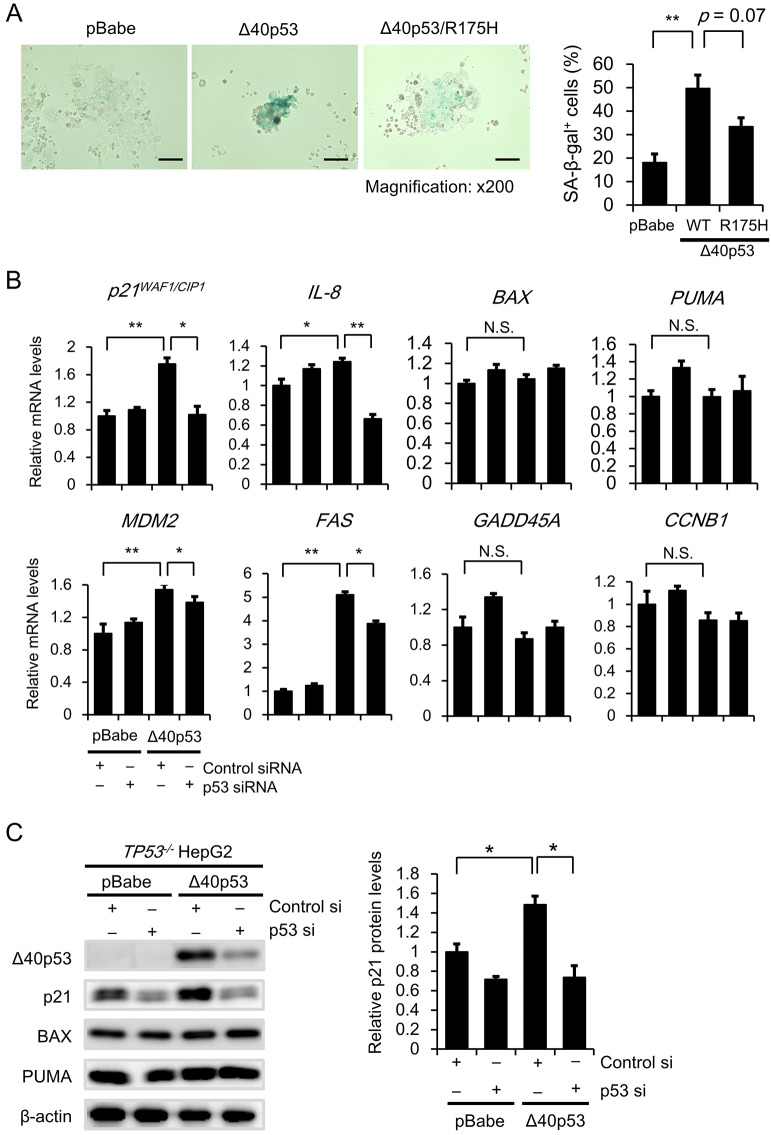
**Effect of Δ40p53 on cellular senescence and expression of p53 target genes.** (A) The cellular senescence of the pBabe, Δ40p53/pBabe, and Δ40p53-R175H/pBabe was examined with HepG2/*TP53^−/−^* cells as described in the legend of Fig. 1E. Bar graphs represent the percentage of SA-β-gal-positive cells (*n*=3). Scale bar: 100 μm. (B,C) pBabe/HepG2/*TP53^−/−^* cells and Δ40p53 (WT)/pBabe/HepG2/*TP53^−/−^* cells were transfected with 50 nM of siRNA specific to *TP53* (p35 si) or nonspecific control siRNA (control si). (B) After 72 h, total RNA was extracted from the cells and mRNA expression levels of *p21*, *IL-8*, *BAX*, *PUMA*, *MDM2*, *FAS*, *GADD45A* and *CCNB1* were determined by qRT-PCR. The primers used for qRT-PCR are presented in Table S3. Relative gene expression levels are presented after normalization to *GAPDH* mRNA expression. The data are expressed as the mean±s.e.m. (*n*=3) relative to the mRNA levels found in the corresponding sample of pBabe/HepG2/*TP53^−/−^* cells, which was arbitrarily defined as 1. (C) After 72 h, protein expression of p21, BAX, PUMA and Δ40p53 were examined by western blot as described in Fig. 1F. After normalization to β-actin protein levels, p21 protein levels are expressed relative to the protein expression in pBabe/HepG2/*TP53^−/−^* cells, which was arbitrarily defined as 1. β-actin was used as an internal control. *P<0.05; **P<0.005.

Because our experimental cell model was generated in the presence of FL-p53, it was difficult to exclude the possibility that Δ40p53 exerts tumor suppressor activity that is dependent upon the expression of FL-p53. To investigate whether Δ40p53 independently exerts tumor suppressor activity, we established a *TP53^−/−^* HepG2 cell clone using the CRISPR/Cas9 system (Fig. 5A). Western blot analysis revealed that FL-p53 was undetectable in the *TP53^−/−^* HepG2 cell clone, whereas it was detected in parental HepG2 cells after 24 h of incubation in the presence or absence of doxorubicin (Fig. 5B). We also confirmed that p53 transcription factor activity was minimal in the *TP53^−/−^* cell clone (Fig. 5C). Thus, we investigated the effect of exogenous Δ40p53 expression on clonogenic activity in the absence of FL-p53. Cells expressing Δ40p53 formed fewer colonies than those expressing control (pBabe) or Δ40p53-R175H (Fig. 5D). Similar results were observed in p53-deleted Hep3B cells (Fig. 5D). Furthermore, the percentage of SA-β-gal-positive cells was significantly higher among those expressing WT-Δ40p53; this increase was partly attenuated in cells expressing Δ40p53-R175H (Fig. 6A). We then examined the effect of Δ40p53 on expression of p53 target genes under *TP53* knockdown. We found that p21 mRNA and protein expression of significantly increased in the Δ40p53/pBabe/HepG2/*TP53^−/−^* cells compared with the pBabe/HepG2/*TP53^−/−^* cells; this increase was attenuated after knockdown of Δ40p53 (Fig. 6B,C). Similarly, mRNA expression level of *IL-8*, *MDM2* and *FAS* significantly increased in Δ40p53 cell clones; these increases were reversed after knockdown of Δ40p53 (Fig. 6C). In addition, we examined the effects of mutant Δ40p53-R175H on *p21*, *CCNB1* and *IL-8* gene expression. We then compared the effects with those obtained in the cells expressing control vector (pBabe) and WT-Δ40p53. qRT-PCR analysis revealed that mRNA expression of *CCNB1* and *IL-8* but not *p21* significantly increased in the cells expressing Δ40p53-R175H (Fig. S4D). These results strongly suggest that Δ40p53 itself exerts tumor suppressor activity.

### Δ40p53 increases the protein levels of FL-p53

**Fig. 7.**
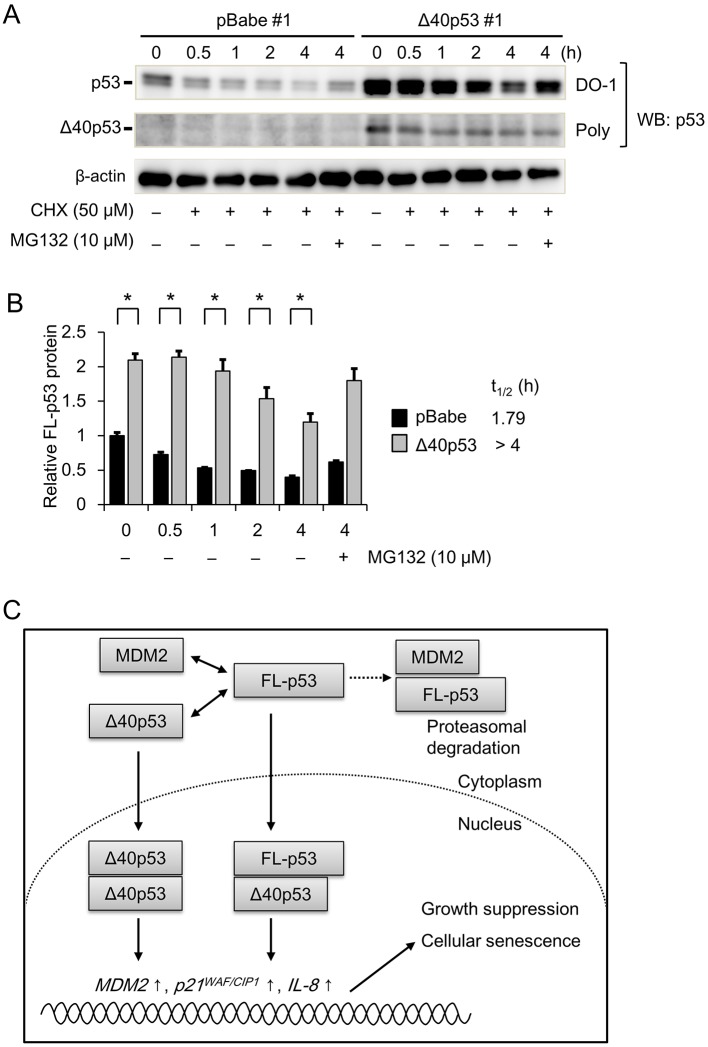
**The effect of Δ40p53 on FL-p53 protein expression.** (A) The pBabe #1 and Δ40p53 #1 (10^5^ cells/well) clones were treated with cycloheximide (CHX; 50 μM) in the presence or absence of the proteasome inhibitor MG132 (10 μM). After treatment for the indicated duration (0, 0.5, 1, 2 or 4 h), the cells were lysed, and the protein lysates were subjected to western blot (WB) analysis to detect the p53 isoforms. A representative western blot result is shown. (B) After normalization to β-actin protein expression, the data from experiments as in A are presented relative to FL-p53 protein expression in the pBabe #1 clone (at 0 h after CHX treatment), which was arbitrarily defined as 1. β-actin was used as an internal control. *P<0.05. The protein half-life (*t*_1/2_) of FL-p53 after CHX treatment is indicated at the right side of the bar graph. (C) A molecular mechanism by which Δ40p53 exerts tumor suppressor activity in HCC cells is proposed. The interaction of Δ40p53 with FL-p53 potentially disturbs the binding of FL-p53 to MDM2, which negatively regulates p53 transcription factor activity through the ubiquitin–proteasome pathway. Thus, Δ40p53 can promote the expression of p53 target genes including *MDM2*, *p21* and *IL-8*, which suppresses tumor growth and induces cellular senescence in HCC cells.

Finally, we examined the effect of Δ40p53 on FL-p53 protein expression using western blot analysis. FL-p53 protein levels were significantly increased in the Δ40p53#1 cells compared to the pBabe#1 cells (Fig. 7A,B). Moreover, the protein levels of FL-p53 were reduced in a time-dependent manner after cycloheximide (CHX) treatment in both pBabe#1 and Δ40p53#1 cells; this reduction was clearly delayed in the Δ40p53#1 cells (Fig. 7B), as indicated by the longer half-life of FL-p53 protein in Δ40p53#1 cells (*t*_1/2_>4 h) compared to pBabe#1 cells (*t*_1/2_=1.26 h). In addition, the reduction in FL-p53 protein levels after CHX treatment was partly attenuated by treatment with the proteasome inhibitor MG132, suggesting that Δ40p53 may preserve the protein levels of FL-p53 by affecting its proteasomal degradation. To further investigate the effect of Δ40p53 on FL-p53-induced anti-tumor activity, we performed a clonogenic assay in the HepG2/*TP53^−/−^* cells expressing pBabe, Δ40p53, FL-p53, and FL-p53 plus Δ40p53 (co-expression). The clonogenic assay revealed that cells expressing FL-p53 formed fewer colonies; this reduction was significantly augmented in cells co-expressing FL-p53 and Δ40p53 (Fig. S4F).

Collectively, these results strongly suggest that Δ40p53 exerts growth suppressive activity and promotes senescence in HCC cells (Fig. 7C).

## DISCUSSION

In the present study, we demonstrate for the first time that Δ40p53 exerts tumor suppressor activity in HCC cells. To investigate the biological function of Δ40p53, we established *TP53* gene-modified cell models, including *TP53^+/Δ40^* HepG2 cells expressing endogenous Δ40p53, parental HepG2 cells expressing exogenous Δ40p53, and CRISPR/Cas9-mediated *TP53^−/−^* HepG2 cells harboring a disrupted *TP53* gene. Takahashi et al. reported that Δ40p53 elicits apoptosis in human melanoma cells (Takahashi et al., 2014). Our data showed that caspase-3 and -7 activity and the annexin V- and PI-positive population were not increased in the *TP53^+/Δ40^* cell clones, indicating that the tumor suppressor activity of Δ40p53 may not be mediated through apoptosis.

HepG2 cells express WT p53, whereas HuH-7 and PLC/PRF/5 cells harbor mutated p53 (HuH-7, *TP53^Y220C^*; PLC/PRF/5, *TP53^R249S^*) (Hsu et al., 1993). Retroviral-induced Δ40p53 expression suppressed colony formation in all three HCC cell lines, in *TP53^−/−^* Hep3B cells, and in genetically engineered *TP53^−/−^* HepG2 cells. These results strongly suggest that Δ40p53 exerts tumor suppressor activity regardless of the FL-p53 status. Furthermore, both endogenous and exogenous Δ40p53 expression significantly suppressed clonogenicity, induced cellular senescence, and upregulated p53 target gene expression in the presence of FL-p53 with or without the *TP53* mutation, strongly suggesting that Δ40p53, which lacks the MDM2-interactive TAD-I domain, may exhibit higher tumor suppressor activity than FL-p53. We detected endogenous expression of Δ40p53 and its upregulation after doxorubicin treatment in both HepG2 and PLC/PRF/5 cells, indicating that Δ40p53 potentially has important anti-tumor effects in HCC cells. Given that Δ40p53 exerted promising anti-tumor activity in HCC cells, it would be interesting to examine whether changes in Δ40p53 expression are related to liver carcinogenesis *in vitro* and/or *in vivo*.

Senescence is a recognized barrier to cellular proliferation (Maier et al., 2004). In this study, there were significantly more SA-β-gal-positive cells among the HepG2 *TP53^+/Δ40^* and Δ40p53/pBabe cell clones compared to the control cell clones. In addition, the increased number of SA-β-gal-positive cells was clearly suppressed by *TP53* knockdown, suggesting that Δ40p53 may play a pivotal role in cellular senescence. *p21*, a well-known p53 target gene, is upregulated during replicative senescence and functions as an inhibitor of proliferation (Lanigan et al., 2011; Brugarolas et al., 1995). We found that the expression levels of both p21 and p53-inducible genes, including *MDM2* and *FAS*, were significantly upregulated in HepG2 *TP53^+/Δ40^* and Δ40p53/pBabe cell clones compared to the control cell clones. These changes in gene expression were abrogated by *TP53* knockdown. Moreover, the tumor suppressor activity of Δ40p53 was partly, but significantly, attenuated in cells expressing mutant Δ40p53-R175H, although the mRNA levels of *p21* and p53-induced genes, including *MDM2* and *FAS*, did not change, providing further experimental evidence that the transcriptional activity of Δ40p53 is closely associated with the its anti-tumor effects. We also observed that cells expressing mutant Δ40p53-R175H protein formed fewer colonies and exhibited increased SA-β-gal positive staining without upregulation of *p21*; however, these levels were reduced compared with cells expressing WT-Δ40p53. Moreover, we found that Δ40p53-R175H significantly increased mRNA expression of *CCNB1* and/or *IL-8*, proteins that lead to a senescence-associated secretory phenotype (SASP). These results suggest that upregulation of *p21* is not always responsible for cellular senescence induced by Δ40p53. In addition, Δ40p53-R175H may exhibit anti-tumor activity by modulating p53 target genes, which are similar to but different from those modulated by WT-Δ40p53. Ohki et al. reported that Δ40p53 induces some, but not all, p53-inducible genes in *TP53*^−/−^ Saos2 cells (Ohki et al., 2007). Thus, further studies, including comprehensive microarray and/or RNA sequencing analyses, may contribute to a better understanding of the molecular basis underlying the role of Δ40p53 and its mutants in tumor suppression and senescence in HCC cells.

Thus far, Δ40p53 has been shown to regulate p53-inducible gene expression in both a positive and negative manner (Yin et al., 2002). Although we did not clarify the interaction of Δ40p53 with FL-p53 or MDM2, our observations that Δ40p53 increased the protein half-life of FL-p53 and augmented the FL-p53-induced anti-tumor activity suggest that Δ40p53 may positively regulate FL-p53 activity in our proposed HCC cell model.

In conclusion, to the best of our knowledge, this study is the first to demonstrate that Δ40p53 exerts tumor suppressor activity and promotes cellular senescence, at least in part, by upregulating p53 target gene expression in HCC cells. Our findings enhance the understanding of the molecular pathogenesis of HCC. Further studies, including *in vivo* experiments, are warranted to investigate the role of Δ40p53 in the pathogenesis of HCC, and such studies should consider the effects of Δ40p53 to advance current therapeutics for patients with HCC.

## MATERIALS AND METHODS

### Cell culture

Human hepatocellular carcinoma cell lines HuH-1 (*TP53*^WT^), HepG2 (*TP53*^WT^), Hep3B (*TP53^−/−^*), HuH-7(*TP53*^Y220C^), and PLC/PRF/5(*TP53*^R249S^) were obtained from Japanese Collection of Research Bioresources Cell Bank (Osaka, Japan). HCC cell lines were maintained in Dulbecco's modified Eagle's medium (DMEM) supplemented with 10% fetal bovine serum (FBS) and penicillin-streptomycin at 37°C in 5% CO_2_ humidified air. Adherent cells were dissociated from a 90-mm dish using trypsin and then seeded in 96-well, 12-well, or 6-well plates for the experiments. 293T cells were from the ATCC (Manassas, VA).

### Reagents

DMEM, β-galactosidase, Crystal Violet, trypsin-EDTA and penicillin-streptomycin solution were purchased from Wako Pure Chemical Industries, Ltd. (Osaka, Japan). Glutaraldehyde was obtained from Sigma-Aldrich (Tokyo, Japan). FBS was obtained from Nichirei Biosciences Inc. (Tokyo, Japan). The mouse anti-human p53 monoclonal antibody (mAb; DO-1) was obtained from Santa Cruz Biotechnologies Inc. (Santa Cruz, CA USA) at was used at 1:400. Annexin V–fluorescein isothiocyanate (FITC) was obtained from MBL (Nagoya, Japan). Propidium iodide (PI) was obtained from Abcam Inc. (Cambridge, MA). Apo-ONE™ Homogeneous Caspase-3/7 Assay Kits were obtained from Promega KK (Tokyo, Japan). All other primary and secondary antibodies used for western blot analysis are described in Table S4. The Cell Proliferation Kit I was purchased from Roche (Tokyo, Japan).

### Targeted deletion of *TP53* exon 2 in the HepG2 cell line

To remove each allele of *TP53* exon 2, a vector targeting exon 2 of *TP53* (a generous gift from the laboratory of Dr Fred Bunz, Radiation Oncology and Molecular Radiation Sciences, John Hopkins University, MD) and the targeted clones were created as previously described (Topaloglu et al., 2005; Weiss et al., 2010; Rago et al., 2007). Knockout of exon 2 of *TP53* was performed using an adeno-associated viral (AAV) vector, as previously described (Weiss et al., 2010). The targeting vector was transduced into cells and antibiotic selection was performed with 800 μg/ml of G418 (Life Technologies) in 96-well plates. Neomycin-resistant colonies were expanded, replicated and pooled, and PCR-based screening was performed as previously described (Konishi et al., 2007). Targeted cells were infected with an adenovirus encoding Cre recombinase to remove the selection cassette. This was followed by single-cell dilutions and then screening by PCR for successful Cre recombination. The primer sequences for PCR are shown in Table S1. To create cell clones carrying homozygous deletions of *TP53* exon 2, *TP53^+/Δ40^* clones were infected with the same AAV targeting vector as shown in Fig. S1A, and concurrently selected with neomycin. During each attempt, ∼1000 drug-resistant cell clones were screened for homologous recombination (HR) events by applying the protocol described above.

### qRT-PCR analysis

HepG2 cells (2×10^5^ cells/well) were seeded in 6-well plates and incubated for 48 h. Total RNA was then extracted using a PureLink^®^ RNA Mini Kit (Life Technologies Japan, Tokyo, Japan), and 2 μg of total RNA was used for cDNA synthesis, which was performed using High Capacity cDNA Reverse Transcription Kits (Life Technologies Japan). qRT-PCR analysis was performed using SYBR Green I, as previously described (Takahashi et al., 2013). Glyceraldehyde-3-phosphate dehydrogenase (*GAPDH*) was used as an internal control. The primers used in this study are described in Table S2.

### Western blot analysis

HepG2 cells were seeded in a 6-well culture plate (2×10^5^ cells/well) and incubated for 48 h. Preparation of cell extracts and western blot analysis were performed as previously described (Hossain et al., 2015). Immune complexes were detected using ImmunoStar LD (Wako) with a LAS-4000 image analyzer (GE Healthcare, Tokyo, Japan). Band intensity was measured using ImageQuant TL software (GE Healthcare). Relative protein levels were calculated after normalization against the internal control β-actin.

### Retrovirus

A fragment containing the Δ40p53 open reading frame (ORF) was amplified from HepG2 cDNA using KOD PlusNeo polymerase (TOYOBO; Tokyo, Japan) and the following primer set: forward, 5′-GGATCCCAAGCAATGGATGAT-3′ and reverse, 5′-TCAGTCTGAGTCAGGCCCTT-3′. A fragment of the Δ40p53 ORF carrying the R175H or R273H mutation was amplified from pCMV-Neo-Bam p53 R175H (plasmid 16436, obtained from Addgene) or pCMV-Neo-Bam p53 R273H (plasmid 16439, obtained from Addgene), respectively. Each fragment was inserted into a pBabe-puro vector or a pLXSN vector (Clontech Laboratories, Inc., Mountain View, CA). The retroviral plasmids were packaged in 293T cells using the pCL10A vector. Viral supernatants were harvested 96 h after transfection and filtered before infection. Experimental HCC cells were infected with retroviruses in the presence of 8 μg/ml Polybrene (Sigma-Aldrich). Antibiotic selection (puromycin; 2 μg/ml for pBabe-puro or neomycin; 800 μg/ml for pLXSN) was begun 48 h after infection and continued for at least 3 days.

### Knockdown of p53

pMKO.1-puro p53 shRNA-2 and pMKO.1-puro GFP shRNA were gifts from William Hahn (Dept. Medical Oncology, Dana-Farber Cancer Institute, MA; Addgene plasmids #10671 and #10675, respectively; Masutomi et al., 2003). To obtain cell clones that exhibited stably decreased p53 expression, either the p53 shRNA vector or the control GFP shRNA vector was introduced into HepG2/*TP53^+/Δ40^* #1 cells. The retroviral plasmids and supernatants were prepared as described above. Following infection and antibiotic selection, single colonies were manually selected, expanded and examined for the expression of p53 at either the mRNA or protein level by using qRT-PCR analysis or western blot analysis, respectively. Transient p53 knockdown was performed using *TP53* siRNA (Cell Signaling Technology).

### Clonogenic assay

HepG2 cell clones were seeded in a 12-well culture plate (200 cells/well) and incubated for 14 days to form colonies, as described previously (Attardi et al., 2004). For retroviral vector-based clonogenic assays, HepG2 cells were seeded into a 6-well culture plate (1×10^5^ cells/well) 1 day before infection. qRT-PCR analysis was performed to quantify the level of retroviral RNA by measuring the copy number of the puromycin N-acetyl-transferase (*PAC* derived from *Streptomyces* strains) gene with a primer set (Table S2). The cells were then infected with the same multiplicity of infection (MOI) for 48 h, which was followed by antibiotic selection, as described above. After incubation for 14 days, the cells were fixed and then stained with 0.1% Crystal Violet in PBS. The number of stained colonies was counted manually.

### Cell proliferation assay

For the MTT cell proliferation assay, HepG2 cells were plated in a 96-well culture plate (10^3^ cells/well) and incubated at 37°C for the indicated durations. After incubation, cell proliferation was assessed by using a Cell Proliferation Kit I (Roche). Absorbance was measured at 595 nm using a SpectraMax M5 spectrophotometer (Molecular Devices, Sunnyvale, CA).

### Annexin V and cellular caspase-3 and -7 activity assay

HepG2 cells were stained with AxV–FITC and PI (10 µg/ml) at room temperature for 15 min. Fluorescent intensities were determined by fluorescence-activated cell sorting (FACS) in a FACSCantoII (BD, Franklin Lakes, NJ), which analyzes 10,000 events (determined by forward and side scatter). The caspase-3 and -7 assay was performed by using an Apo-ONE™ Homogeneous Caspase-3/7 Assay Kit (Promega) according to the manufacturer's instructions. Fluorescence intensity (499 nm excitation and 521 nm emission) was measured using a SpectraMax M5 spectrophotometer (Molecular Devices).

### Cell cycle analysis

HepG2 cells were seeded in a 6-well culture plate (10^5^ cells/well) and incubated for 24 h. To synchronize the cell cultures, the cells were rinsed in PBS and serum-free DMEM was then added. After serum starvation for 48 h, the cells were released into the cell cycle by the addition of FBS. For FACS analysis, the cells were detached using trypsin at 24 h after serum treatment and fixed in ice-cold 70% ethanol overnight. After fixation, the cells were treated with RNase A (100 μg/ml) and stained with PI (100 μg/ml). The percentages of cells in the sub-G1, G1, S, and G2–M phases were measured using FlowJo software (Tree Star Inc., Ashland, OR).

### *TP53* knockout using the CRISPR-Cas9 system

The CRISPR-Cas9 system was used to disrupt the expression of the *TP53* gene, as described previously (Ran et al., 2013). pSpCas9(BB)-2A-Puro (PX459) was a gift from Feng Zhang (Broad Institute of MIT and Harvard, MA; Addgene plasmid #48139) (Ran et al., 2013). In brief, a single guide RNA (sgRNA) sequence was selected using Optimized CRISPR Design (http://crispr.mit.edu/). The sgRNA sequence for *TP53* was 5′-ATCCATTGCTTGGGACGGCA-3′ (hereafter called *TP53* sgRNA). The plasmid expressing human Cas9 and the *TP53* sgRNA were prepared by ligating oligonucleotides into the BbsI site of PX459 (*TP53*/PX459). To examine the efficacy of the sgRNA, we used pCAG-EGxxFP, which was a gift from Masahito Ikawa (Osaka University, Japan; Addgene plasmid #50716) (Mashiko et al., 2013). An ∼500 bp long genomic fragment containing the sgRNA target sequence was amplified using PCR and cloned between the EGFP fragments of pCAG-EGxxFP (*TP53*/pCAG-EGxxFP). We then co-transfected 293T cells with *TP53*/PX459 and *TP53*/pCAG-EGxxFP. After 4 days of transfection, the efficacy of HR of the *EGFP* gene was evaluated using fluorescence microscopy analysis (BZ-9000, KEYENCE, Osaka, Japan). To establish a *TP53^−/−^* clone, HepG2 cells (1×10^6^ cells/dish) were seeded in a 10 cm dish. The cells were then transfected with 10 μg of *TP53*/PX459 using Lipofectamine 3000 (Life Technologies). Antibiotic selection (puromycin; 2 μg/ml) was begun 72 h after infection and continued for at least 3 days. A single clone was selected, expanded, and then used for biological assays. For sequence analysis of the *TP53* gene, the following primer set was used: 5′-CAGCCATTCTTTTCCTGCTC-3′ and 5′-TGCCCTGGTAGGTTTTCTGG-3′.

### Statistical analysis

At least three independent experiments and three replications per experiment were performed. The results are expressed as the mean±s.e.m. Statistical significance between groups was determined using one-way ANOVA and Dunnett's comparison. Statistical analyses were performed using SPSS 23.0 (SPSS Inc; Chicago, IL).
